# Aryl hydrocarbon receptor-dependent upregulation of Cyp1b1 by TCDD and diesel exhaust particles in rat brain microvessels

**DOI:** 10.1186/2045-8118-8-23

**Published:** 2011-08-25

**Authors:** Aude Jacob, Anika MS Hartz, Sophie Potin, Xavier Coumoul, Salah Yousif, Jean-Michel Scherrmann, Björn Bauer, Xavier Declèves

**Affiliations:** 1 Neuropsychopharmacologie des addictions (CNRS UMR 8206), Université Paris Descartes, Sorbonne Paris Cité, Faculté de Pharmacie, Paris, France; 2 INSERM U705, Neuropsychopharmacologie des addictions, Paris, France; 3 Department of Biochemistry and Molecular Biology, Medical School, University of Minnesota, Duluth, USA; 4 Department of Pharmaceutical Sciences, College of Pharmacy, University of Minnesota, Duluth, USA; 5 Brain Barriers Research Center, College of Pharmacy, University of Minnesota, Duluth, USA; 6 INSERM UMR-S747, Toxicologie, pharmacologie et signalisation cellulaire, Université Paris Descartes, 45 rue des Saints-Pères 75006 Paris, France

**Keywords:** Aryl hydrocarbon Receptor, Cyp1b1, TCDD, diesel exhaust particles, brain microvessels

## Abstract

**Background:**

AhR activates the transcription of several target genes including CYP1B1. Recently, we showed *CYP1B1 *as the major cytochrome P450 (CYP) enzyme expressed in human brain microvessels. Here, we studied the effect of AhR activation by environmental pollutants on the expression of Cyp1b1 in rat brain microvessels.

**Methods:**

Expression of AhR and Cyp1b1 was detected in isolated rat brain microvessels. AhR was immunovisualised in brain microvessel endothelial cells. The effect of AhR ligands on Cyp1b1 expression was studied using isolated brain microvessels after *ex vivo *and/or *in vivo *exposure to TCDD, heavy hydrocarbons containing diesel exhaust particles (DEP) or Δ^9^-tetrahydrocannabinol (Δ^9^-THC).

**Results:**

After *ex vivo *exposure to TCDD (a highly potent AhR ligand) for 3 h, *Cyp1b1 *expression was significantly increased by 2.3-fold in brain microvessels. A single i.p. dose of TCDD also increased *Cyp1b1 *transcripts (22-fold) and Cyp1b1 protein (2-fold) in rat brain microvessels at 72 h after TCDD. Likewise, DEP treatment (*in vivo *and *ex vivo*) strongly induced Cyp1b1 protein in brain microvessels. DEP-mediated Cyp1b1 induction was inhibited by actinomycin D, cycloheximide, or by an AhR antagonist. In contrast, a sub-chronic *in vivo *treatment with Δ^9^-THC once daily for 7 seven days had no effect on *Cyp1b1 *expression

**Conclusions:**

Our results show that TCDD and DEP strongly induced Cyp1b1 in rat brain microvessels, likely through AhR activation.

## Background

The blood-brain barrier (BBB) is a dynamic neurovascular unit composed of three main cell types: brain microvessel endothelial cells sealed by tight junctions, astrocyte end-foot processes ensheathing microvessels, and pericytes sharing the basal membrane with endothelial cells. Due to tight junctions limiting paracellular diffusion and the presence of drug efflux transporters and drug-metabolizing enzymes, the BBB is both a physical and a metabolic barrier that selectively controls brain penetration of xenobiotics. Recently, we showed that CYP1B1, a cytochrome P450 (CYP) enzyme, was the most highly expressed CYP gene in human brain microvessels [1]. Expression of genes encoding CYP enzymes is regulated in several peripheral tissues by transcription factors such as the pregnane-X-receptor (PXR), the constitutive androstane receptor (CAR), and the aryl hydrocarbon receptor (AhR) [2]. AhR is a cytosolic ligand-dependent transcription factor of the bHLH-PAS (basic helix-loop-helix/Per-Arnt-Sim) superfamily. AhR is activated by binding of several classes of structurally different aromatic compounds and environmental pollutants such as polycyclic aromatic hydrocarbons (PAHs) and halogenated aromatic hydrocarbons (HAHs) like 2,3,7,8-tetrachlorodibenzo-p-dioxin (TCDD, dioxin) [3]. Briefly, upon ligand binding in the cytosol, AhR translocates into the nucleus where it heterodimerizes with its partner aryl hydrocarbon nuclear translocator (ARNT). The AhR/ARNT complex activates in turn xenobiotic response elements (XRE) in the promoter region of several AhR target genes, thus increasing their transcription [4,5]. AhR target genes include several genes encoding CYP enzymes such as CYP1A1, CYP1A2 and CYP1B1 [6,7], as well as ATP-binding cassette (ABC) transporters such as ABCG2 [8] and genes involved in cell cycle regulation, cell proliferation, inflammation, and apoptosis [9,10]. High levels of *AhR *mRNA were detected in human brain microvessels and in the hCMEC/D3 human cerebral microvascular endothelial cells, a promising *in vitro *model of the human BBB [1,11]. Furthermore, TCDD, the most potent AhR activator, highly induced the expression of CYP1B1 and CYP1A1 in hCMEC/D3 cells [11]. Consistent with this, AhR agonists increased Cyp1a1 and Cyp1b1 in many brain regions in rat and in blood-brain interfaces of mice and rats [12,13]. TCDD has also been shown to induce both Cyp1a1 and Cyp1b1 in cultured murine cerebral vascular endothelial cells and astrocytes [14]. Regarding Cyp1b1, this enzyme has been demonstrated to metabolise various endogenous compounds like retinol, arachidonic acid, melatonin and estrogens [7,15]. Moreover, Cyp1b1 also selectively bioactivates various PAHs (benzo[a]pyrene, DMBA, anthracenes) into intermediates that are DNA-reactive [16]. All these studies suggest that AhR control the expression of CYP1B1 at the BBB.

First, this work aimed to examine TCDD-mediated activation of AhR on Cyp1b1 expression *in vivo *in rat brain microvessels. In addition, we studied the effect of two other potential AhR ligands: Δ^9^-tetrahydrocannabinol (Δ^9^-THC) and diesel exhaust particles (DEP). Δ^9^-THC, the psychoactive component of marijuana, has been discussed as a potential AhR ligand due to its ability to induce *Cyp1a1 *expression in a murine hepatoma cell line [17]. DEP are complex environmental toxicants containing heavy hydrocarbons derived from fuel like PAHs and HAHs that people are exposed to daily and known to activate AhR [18,19].

## Methods

### Reagents and equipment

RNA extraction kits were purchased from Qiagen GmbH (Hilden, Germany). RT-PCR reagents were purchased from Invitrogen (Invitrogen, France). Primers were synthesized by Invitrogen Life Technologies (Invitrogen, France). The LC Fast Start DNA Master SYBR Green I kit was purchased from Roche Diagnostics (Meylan, France), and the Power SYBR Green PCR Master Mix was from Applied Biosystems (Foster City, CA, USA). 2,3,7,8-tetrachlorodibenzo-p-dioxin (TCDD, dioxin, 50 μg/mL in DMSO) was obtained from LGC Promochem (Molsheim, France) and Δ^9^-THC (27 mg/mL in ethanol) was from Sigma-Aldrich (Saint Quentin Fallavier, France). Antibodies and supplies used for western blotting were: polyclonal rabbit anti-rat Cyp1a1, polyclonal rabbit anti-Cyp1b1, monoclonal mouse anti-AhR, and monoclonal mouse anti-*β*-actin antibodies (Abcam, Cambridge, UK and Abcam, Cambridge, MA, USA), horseradish peroxidase-conjugated monkey anti-rabbit secondary antibody and horseradish peroxidase-conjugated monkey anti-mouse secondary antibody (Amersham, Buckinghamshire, UK), and Alexa Fluor^® ^488 goat anti-mouse IgG (H+L) antibody (Invitrogen, Carlsbad, CA, USA). Proteins were detected using SuperSignal^® ^West Pico Chemoluminescent Substrate (Pierce, Rockford, IL, USA). Diesel exhaust particles (DEP) were obtained from NIST [20] (Gaithersburg, MD, USA). Carbon black (CB) mock particles were a kind gift from Degussa Corporation (Akron, OH, USA). AhR antagonist (CH-223191) [21] and IgG control antibody were from Calbiochem-Novabiochem (La Jolla, CA, USA). Protein A/G Beads were from Pierce (Rockford, IL). Other chemicals and reagents were purchased from Sigma-Aldrich (France and St. Louis, MO, USA) or Invitrogen (Cergy-Pontoise, France). Equipments used were: a nucleic acid spectrophotometer (Nanodrop ND-1000, NanoDrop Technologies, USA), a programmable thermal cycler (PTC-100 programmable thermal controller, MJ research Inc., USA), a Light Cycler thermal cycler (Light-Cycler^® ^instrument, Roche Diagnostics), and a 7900 HT Real-Time PCR Detection System (Applied Biosystems, Foster City, CA).

### Animal treatments

All animal experiments were based on the same experimental setup. For treatment with TCDD and Δ^9^-THC, male Sprague-Dawley rats (230-250 g) were obtained from Charles River (L'arbresle, France). For treatment with DEP, male CD^® ^IGS Sprague-Dawley rats (275-300 g) were obtained from Charles River (Portage, MI, USA). Rats were housed in groups of four animals per cage under standard 12:12-hour light/dark conditions (light from 8:00 a.m. to 8 p.m.) in a temperature- and humidity-controlled room. Animals had access to food and water *ad libitum*. Before using rats for experiments, they were allowed to adapt to the animal facility for 3-5 days.

The care and treatment of animals (TCDD and Δ^9^-THC treatment) were in accordance with standards and guidelines approved by the European Communities Council Directive (86/609/EEC). Animal protocols were approved by the Institutional Animal Care and Use Committees of the EPA, RTP, NC, and the University of Minnesota and were in accordance with AAALAC regulations and the Guides to Animal Use of the University of Minnesota and NIH animal guidelines.

For *in vivo *TCDD exposure, animals were dosed with a single i.p of TCDD (25 μg/kg body weight) or control (1:9 DMSO: Corn Oil) and sacrificed 72 h after the TCDD or control injection as previously described [22,23].

For *in vivo *Δ^9^-THC exposure, Δ^9^-THC (10 mg/kg body weight) or control (1:1:18 ethanol: cremophor: saline) were administered i.p once daily for 7 days. This *in vivo *dosing protocol was chosen for several reasons: (1) Δ^9^-THC has a half-life of about 25-36 h, which is long enough for a once daily administration, (2) Δ^9^-THC is likely a less potent AhR activator compared to TCDD, (3) the 7-day Δ^9^-THC dosing scheme is currently used in our laboratory, and we observed that it causes an addiction-like behavioral effect (unpublished data). Rats were sacrificed 12 h after the last Δ^9^-THC or control injection.

For *in vivo *DEP exposure, diesel engine exhaust was generated by operating a 30-kW (40 hp) four-cylinder indirect injection Deutz diesel engine (BF4M1008) and was collected as previously described [24]. Rats in inhalation chambers were exposed to 0.5 mg/m^3 ^and 2 mg/m^3 ^of this diesel engine exhaust for 5 h/day, 5 days/week for 4 consecutive weeks. Inhalation fumes contained diesel exhaust engine gas as well as diesel exhaust particulate matter. These DEP inhalation doses have been used in previous studies and are based on DEP concentrations found in ambient air during heavy traffic [25,26].

### Preparation of DEP working suspension

DEP working suspension using diesel exhaust particles (SRM 2975) that were derived from diesel-powered forklifts was prepared as previously described [27]. Briefly, 2 mg of DEP was suspended in 10 mL PBS buffer, vortexed, and sonicated for 30 min at 25°C using an ultrasonic processor (Bransonic^®^, Model 3510, Branson Ultrasonic Cooperation, Danbury, CT, USA). The suspension was filtered through a 0.22-μm filter (MillexGS; Millipore, Billerica, MA, USA).

### Isolation of rat brain microvessels

All steps in the isolation of brain microvessels were carried out at 4°C. Rats treated with TCDD, Δ^9^-THC or DEP were anesthetized with isoflurane and sacrificed by decapitation after the last injection or euthanized by CO_2 _inhalation and decapitated. Brains were immediately removed and placed in ice-cold HBSS. Cerebella, meninges, brainstems, and large superficial blood vessels were removed. Rat cortex microvessels were isolated according to a protocol that results in the lowest contamination with astrocyte and neuron mRNA and the highest yield of endothelial cells mRNA [28]. Isolated brain microvessels were also free from red blood cells, other contaminating cells such as microglia, and cell debris [27]. Briefly, cortices were minced and homogenized with a Potter-Thomas homogenizer (Konte Glass, Vineland, NJ, USA). The resulting homogenate was centrifuged at 1000 g for 10 min, and the microvessel-enriched pellet was suspended in 17.5% dextran and centrifuged for 15 min at 4400 g at 4°C in a swinging bucket rotor. The resulting pellet was suspended in HBSS containing 1% BSA, while the supernatant containing a layer of myelin was centrifuged once more. The resulting microvessel suspension was passed through a 100 μm nylon mesh, and the filtrate was then passed through a 20 μm nylon mesh. Microvessels retained by the nylon 20 μm mesh were immediately collected and used for experiments or frozen at -80°C.

For *ex vivo *experiments, freshly isolated brain microvessels from naïve rats were incubated with different concentrations of either TCDD (25 nM) or DEP (0, 5, 50, 200 μg/mL) as previously described [27].

### RNA extraction and reverse transcription

Total RNA was extracted from tissues and organs of each rat. Total RNA from isolated brain microvessels was obtained by lysing the surrounding basal lamina with proteinase K and then extracting total RNA using the RNeasy Fibrous Tissue Micro kit according to the manufacturer's instructions. RNA samples were purified from contaminating genomic DNA by treatment with DNase (RNase-Free DNase Set, Qiagen SA). RNA concentration and purity were assessed spectrophotometrically at 260 nm using a Nanodrop^® ^spectrophotometer; RNA integrity was assessed by electrophoresis on a 0.8% agarose gel. One μg of total RNA was reverse transcribed into cDNA in a final volume of 20 μL. The mixture consisted of 1 μg total RNA, 500 μM of each dNTP, 10 mM DTT, 1.5 μM random hexa-nucleotide primers, 20 U RNAse in ribonuclease inhibitor, and 100 U SuperScript II reverse transcriptase. Hexamers were annealed at 25°C for 10 min, products were extended at 42°C for 30 min, and the reaction was terminated by heating to 99°C for 5 min before being quick-chilled to 4°C and stored at -80°C.

### Non-quantitative and quantitative RT-PCR (q-PCR)

RT-PCR for *AhR *and *Cyp1b1 *was performed with *Taq *DNA Polymerase from Promega (Madison, WI, USA) using primers for rat *AhR *and *Cyp1b1 *(Table 1) that were custom-synthesized by Qiagen Operon (Alameda, CA, USA). PCR products were resolved on a 2% agarose gel at 100 V for 75 min.

**Table 1:** Sequences of primers used for RT-PCR and q-PCR

Gene	Forward primer (5'-3')	Reverse Primer (5'-3')	Length (bp)	GenBank accession*
*β-Actin*	CTGGCCCGGACCTGACAGA	GCGGCAGTGGCCATCTCTC	132	NM_031144
*AhR^a^*	CTCCCTCCACAGTTGGCTTTGTTTG	GATTCTGCGCAGTGAAGCATGTCAG	233	NM_013149
*Cyp1a1*	AACCCACACCTGTCACTGA	CTGGTGAAACAGGGGGAT	132	NM_012540
*Cyp1b1*	GCTTGCCAGTGAGAGAGG	TTCTCAAGAATGAGCGGAA	135	NM_012940
*Cyp1b1^a^*	GCAGATCAACCGCAACTTCAGCAAC	GTCTGTAATAGTGGCAGGCACATCC	193	NM_012940

*^a ^*for RT-PCR

* http://www.ncbi.nlm.nih.gov/

The effect of Δ^9^-THC and TCDD on the expression of *Cyp1a1 *and *Cyp1b1 *was investigated by quantitative real-time PCR (q-PCR). ß-actin was used as an endogenous reference for normalizing target gene mRNA. Gene expression was evaluated by q-PCR as previously described [28]. Primers were designed using OLIGO 6.42 software (Medprobe, Norway) (Table 1). PCR reactions were performed on a Light-Cycler^® ^instrument using the LC-FastStart DNA Master SYBR Green I kit. cDNAs from either control or treated rats were used to generate external calibration standards for each gene. The PCR reaction mixture consisted of 1 μL LC-FastStart DNA Master SYBR Green mix, 1.2 μL of 10 mM MgCl_2_, 0.5 μL of each upper and lower primer (final concentration 0.5 μM), and 1.8 μL water. The cDNAs were diluted 40-fold, and 5 μL aliquots were mixed with an equal volume of PCR mixture to yield a final volume of 10 μL. The thermal cycling conditions were 8 min at 95°C followed by 40 amplification cycles at 95°C for 5 s, 64°C for 5 s, and 72°C for 5 s. A target gene was considered to be easily quantifiable when the Ct that was obtained for the less diluted cDNA (1/20) sample was lower than 30. A Ct value of 32 was set as the detection limit. A calibration curve using serial dilutions of cDNA standard was used to determine the relative expression of *Cyp1a1 *and *Cyp1b1 *genes in control (vehicle) and TCDD-treated rat (n = 8 rats per group) and in control (vehicle) and Δ^9^-THC-treated rat (n = 12 rats per group). The (fold) change was expressed by the ratio (gene of interest/*ß*-actin)_treated_/(gene of interest/*ß*-actin)_control._

### Immunofluorescence

Freshly isolated brain microvessels were transferred to glass cover slips and fixed for 15 min with 3% paraformaldehyde/0.2% glutaraldehyde at room temperature. After washing with PBS, microvessels were permeabilised for 20 min with 1% (v/v) Triton X-100 in PBS and subsequently blocked with 1% BSA in PBS. Microvessels were incubated overnight at 4°C with the primary antibody to AhR (1:100). After washing with 1% BSA, microvessels were incubated for 1 h at 37°C with Alexa Fluor^® ^488-conjugated secondary IgG (1:500, 4 μg/mL; Invitrogen, Eugene, OR, USA); negative controls were incubated with secondary antibody only. Nuclei were counterstained in blue with 2.5 μg/mL DAPI for 15 min. AhR staining was visualised in green using confocal microscopy (Nikon C1 LSC microscope unit, Nikon TE2000 inverted microscope, 40x oil immersion objective, NA 1.3, 488 nm line of an argon laser, 402 nm line of a solid state UV laser; Nikon Instruments Inc., Melville, NY, USA).

### Immunoprecipitation

Isolated brain microvessels were homogenized in lysis buffer (Sigma, St. Louis, MO, USA) containing Complete^® ^protease inhibitor (Roche, Mannheim, Germany). The lysate was cleared by centrifugation at 10 000 g for 15 min at 4°C. Identical amounts of pre-cleared microvessel lysates (50 μg of protein) were immunoprecipitated with 5 μg AhR antibody, 5 μg IgG antibody (IgG control), or antibody-free PBS buffer (negative control) by overnight incubation at 4°C. The immune complexes were precipitated with Protein A/G beads (Pierce, Rockford, IL, USA) and washed 3 times with RIPA buffer (Sigma, Dedham, MA, USA) and once with PBS. Immunoprecipitated proteins were eluted with LDS buffer (Invitrogen, Carlsbad, CA, USA) and analyzed by western blotting.

### Western blotting analysis

For Cyp1b1, Cyp1a1, and AhR western blots, brain microvessels were homogenized in lysis buffer (Sigma, St. Louis, MO, USA) containing Complete^® ^protease inhibitor (Roche, Mannheim, Germany) and centrifuged at 10 000 g for 15 min. Denucleated supernatants were used as microvessel lysates, and protein concentrations were determined. Western blots were performed using the Invitrogen NuPage™ Bis-Tris electrophoresis and blotting system (Invitrogen, Carlsbad, CA, USA). After protein transfer, blotting membranes were blocked and incubated with primary antibody (AhR 1:100, Cyp1b1 1:500 (1 μg/mL), Cyp1a1 1:1000, *β*-actin 1:1000 (1 μg/mL)). Membranes were washed and incubated with horseradish peroxidase-conjugated ImmunoPure^® ^secondary IgG (1:15,000; Pierce, Rockford, IL, USA) for 1 h. Proteins were detected using SuperSignal^® ^West Pico Chemoluminescent Substrate (Pierce, Rockford, IL, USA), and bands were visualised and recorded using a BioRad Gel Doc 2000™ gel documentation system (BioRad, Hercules, CA, USA). Rat Cyp1a1 supersomes™ and Cyp1b1 microsomes were supplied by BD Biosciences (Woburn, MA, USA) and used as positive controls for Cyp1a1 and Cyp1b1, respectively.

### Statistical analysis

Statistical analyses were done with GraphPad Prism^® ^4.0 software (GraphPad Software Inc., San Diego, CA, USA). Data for qPCR are expressed as means ± SD. Student's unpaired *t*-tests were performed to identify significant differences between rats treated with TCDD or Δ^9^-THC and control-treated rats. All the tests were two-tailed and statistical significance was set at *p *< 0.05. Data received from optical density measurements from western blots are means ± SEM.

## Results

### AhR expression in different rat tissues and its localization in isolated rat brain microvessels

**Figure 1:**
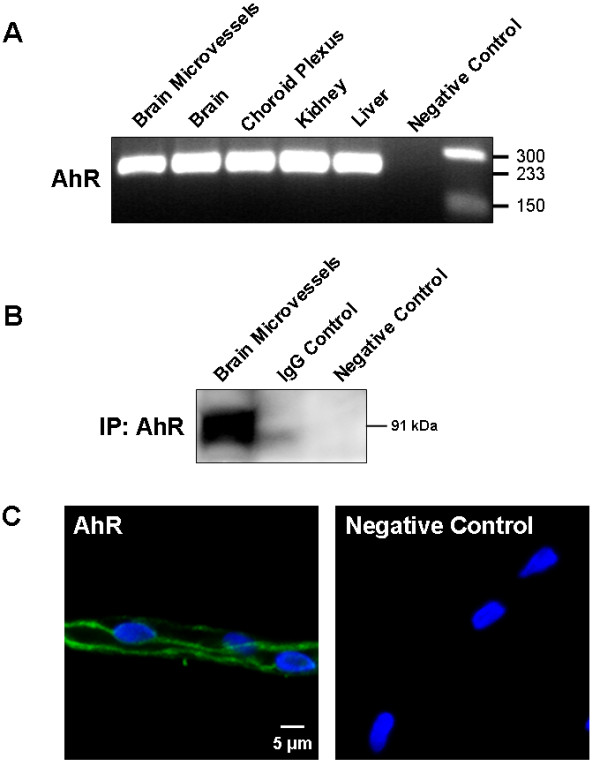
**AhR expression in different rat tissues and AhR immunolocalization in isolated rat brain microvessels**. (A) RT-PCR of liver, kidney, choroid plexus, total brain, and rat brain microvessels for rat *AhR*. The RT-PCR shows a signal for *AhR *mRNA at 233 bp. (B) Western blot of immunoprecipitated AhR from rat brain microvessels. (C) Representative image of a brain microvessel immunostained for AhR (green); nuclei were counterstained with DAPI (blue).

*AhR *transcripts (233 bp amplicon) were detected in total brain, brain microvessels, choroid plexus and peripheral tissues (Figure 1A). AhR protein was also detected after immunoprecipitation and western blotting in brain microvessels, which showed a strong signal for AhR (Figure 1B). Importantly, immunoprecipitations without AhR antibody (negative control) or using an IgG control antibody (IgG control) instead of anti-AhR antibody showed no band demonstrating the specificity of the AhR signal. Using immunostaining we localised AhR in the cytoplasm of brain microvessel endothelial cells (Figure 1C), which is consistent with the location of this transcription factor [9,10]. Thus, AhR is expressed at both the mRNA and protein levels in rat brain microvessels.

### Effect of TCDD and Δ^9^-THC on CYP expression in isolated brain microvessels

**Figure 2:**
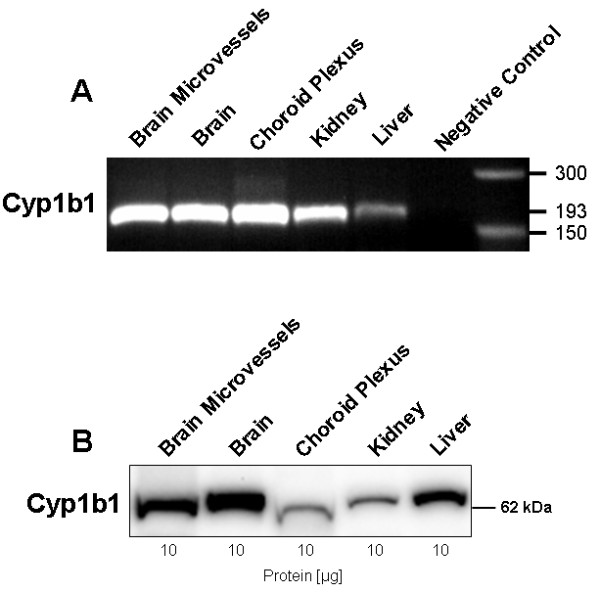
**Gene and protein expression of Cyp1b1 in different rat tissues**. (A) RT-PCR of *Cyp1b1 *transcript levels in liver, kidney, choroid plexus, total brain, and isolated brain microvessels. A signal was detected at 193 bp. (B) Western blot showing Cyp1b1 protein expression at 62 kDa.

To demonstrate expression of Cyp1b1, we first performed both RT-PCR and western blotting. High levels of Cyp1b1 transcript (193 bp amplicon, Figure 2A) and protein were detected in brain microvessels, total brain, liver, choroid plexus and kidney (Figure 2B). The apparent molecular weight of Cyp1b1 protein was determined by digital analysis to be 62 kDa, a value that is consistent with the calculated molecular weight of Cyp1b1 at 60.5 kDa.

**Figure 3:**
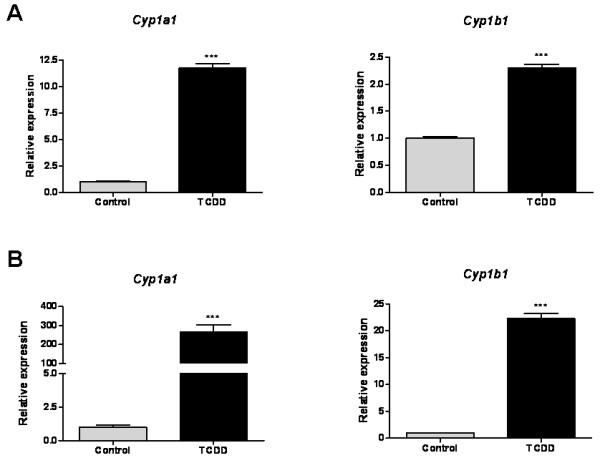
**Effect of TCDD on *Cyp1a1 *and *Cyp1b1 *mRNA levels in isolated rat brain microvessels**. (A) Freshly isolated rat brain microvessels from naïve rats were incubated *ex vivo *for 3 h with 25 nM TCDD (n = 4 rats per group). (B) Rats were dosed with a single i.p. dose of 25 μg/kg TCDD and brain microvessels were isolated at 72 h (n = 8 rats per group). Data are shown as relative expression of *Cyp1a1 *or *Cyp1b1 *mRNA in treated and control rats using q-PCR. Results are expressed as means ± SD ***p < 0.001 (Student's t-test).

To test if AhR induces *Cyp1a1 *and *Cyp1b1 *genes in isolated brain microvessels, we first incubated *ex vivo *brain microvessels from naïve rats with 25 nM TCDD, a highly potent AhR ligand, and determined the expression of *Cyp1b1 *and *Cyp1a1*, two well-known AhR target genes. In control brain microvessels, *Cyp1a1 *mRNA was about 60-fold less abundant than that of *Cyp1b1*. *Cyp1b1 *and *Cyp1a1 *mRNA expression levels in brain microvessels were significantly increased by 2.5-fold and 11-fold as early as 3 h after *ex vivo *TCDD exposure, respectively (Figure 3A).

To test the effect of TCDD on Cyp1a1 and Cyp1b1 *in vivo*, we dosed animals with TCDD (one dose of 25 μg/kg i.p.) and measured *Cyp1a1 *and *Cyp1b1 *transcript levels in isolated brain microvessels with q-PCR at 72 h after TCDD administration. TCDD substantially increased *Cyp1b1 *and *Cyp1a1 *mRNA levels in isolated brain microvessels by 22-fold and 260-fold, respectively (Figure 3B). In some vehicle-treated samples, *Cyp1a1 *transcripts were not detectable and relative expression was therefore calculated using the lowest standard calibration curve.

Δ^9^-THC has been discussed as a potential AhR ligand due to its ability to induce *Cyp1a1 *expression in a murine hepatoma cell line [17]. Therefore, we studied whether sub-chronic treatment with Δ^9^-THC induces Cyp1b1 and Cyp1a1 in rat brain microvessels. Importantly, Δ^9^-THC did not change *Cyp1b1 *expression (1.0 ± 0.12 in untreated rats *versus *1.1 ± 0.18 in Δ^9^-THC-treated rats, n = 12 per group, data not shown). *Cyp1a1 *transcripts were not quantified in brain microvessels from control- and Δ^9^-THC-treated rats.

**Figure 4:**
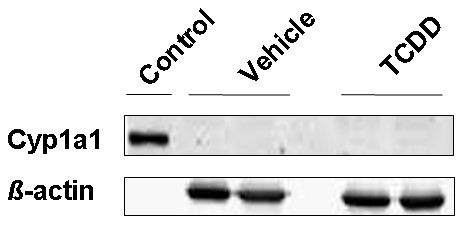
**Cyp1a1 protein expression in brain microvessels of TCDD-treated rats**. Rats were dosed with a single dose of TCDD (25 μg/kg i.p.) and brain microvessels were isolated after 72 h for Cyp1a1 analysis by Western blotting; rat Cyp1a1 supersomes were used as positive control for Cyp1a1 expression.

**Figure 5:**
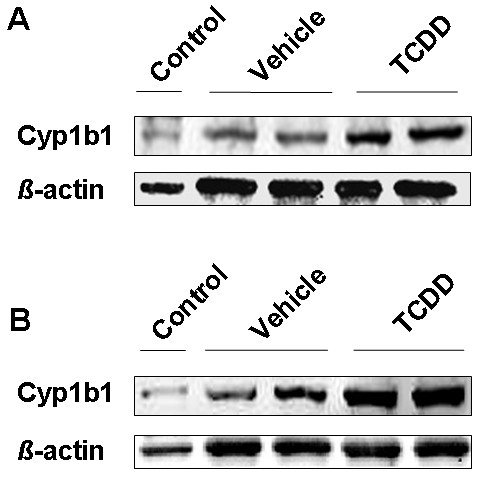
**Cyp1b1 protein expression in brain microvessels of TCDD-treated rats**. Rats were administered a single dose of TCDD (25 μg/kg i.p.), and brain microvessels were isolated 12 h (A) and 72 h (B) after dosing. Rat microsomes were used as positive control for Cyp1b1 expression. The amount of Cyp1b1 in each sample was measured by densitometric analysis and normalized to β-actin.

Since TCDD strongly increased mRNA expression of *Cyp1b1 *and *Cyp1a1 *in isolated brain microvessels, we measured Cyp1a1 protein levels 72 h after TCDD administration. Cyp1a1 protein was not detected in either TCDD-treated or untreated rats. However, Cyp1a1 was present in rat supersomes™ (positive control) at the correct molecular weight (59 kDa; Figure 4). In contrast, 12 h (Figure 5A) and 72 h (Figure 5B) after TCDD dosing, Cyp1b1 expression was increased by 80 ± 13% and 100 ± 8% (SEM) respectively in TCDD-treated rats compared to control rats.

### Effect of DEP exposure on Cyp1b1 expression in isolated rat brain microvessels

**Figure 6:**
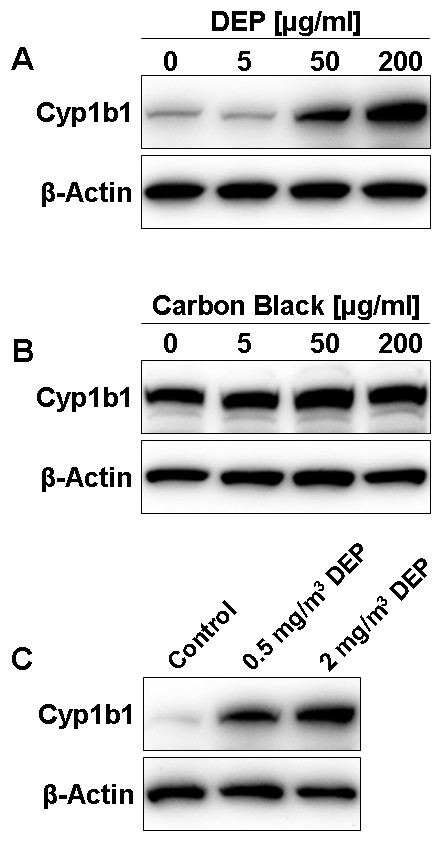
**Effect of DEP exposure on Cyp1b1 expression in isolated rat brain microvessels**. Western blots showing Cyp1b1 protein expression; β-actin was used as loading control. (A) *Ex vivo *DEP exposure of brain microvessels increased Cyp1b1 in a concentration-dependent manner. (B) Carbon black particles were used as a negative control for particulate matter and did not have any effect on Cyp1b1 protein levels in brain microvessels. (C) Cyp1b1 protein expression was increased in brain microvessels from rats that were exposed *in vivo *to 0.5 and 2 mg/m^3 ^diesel engine exhaust containing DEP for 5 h/day, 5 days/week for 4 consecutive weeks. Brain capillaries were pooled from 6 rats per treatment group.

Since Cyp1a1 was not detected at the protein level after TCDD exposure, we focused on AhR-dependent regulation of Cyp1b1 in the following studies. Diesel exhaust particles (DEP) are a real environmental toxicant that billions of people are exposed to on a daily basis [18]. Once inhaled, DEP can enter the circulation and translocate to tissues throughout the body. Through this route, DEP can reach the brain microvasculature and studies showed that DEP even enter the brain [29-31]. DEP consist of a carbon core with adsorbed organic chemicals such as PAHs and HAHs that are known AhR activators [18,19]. Thus, in addition to the DEP itself, the AhR-activating chemicals that are adsorbed to the carbon core also enter the blood-stream and reach the blood-brain barrier where they affect expression of proteins [27]. Here we show that exposing rat brain microvessels to DEP for 6 h *ex vivo *increased Cyp1b1 protein in a concentration-dependent manner up to 310 ± 16% (SEM) of controls at 200 μg/ml DEP (Figure 6A; data are means from 6 densitometric measurements; brain capillaries used for western blots were pooled from 10 rats). In contrast, carbon black mock particles that are used as a negative control for particulate matter did not cause such an increase in Cyp1b1 protein in brain microvessels (Figure 6B). Note that the difference in band intensities for the controls in Figures 6A and 6B is not due to different expression levels, but to different exposure times. Figure 6C shows that Cyp1b1 protein expression was also significantly increased in brain microvessels from rats that were exposed to 0.5 and 2 mg/m^3 ^diesel engine exhaust containing DEP for 5 h/day, 5 days/week for 4 consecutive weeks (0.5 mg/m^3^: 447 ± 26%, 2 mg/m^3^: 538 ± 40% of controls; data are means ± SEM from 5 densitometric measurements; brain capillaries used for western blots were pooled from 6 rats per treatment group). Note that these DEP doses are based on DEP concentrations found in ambient air during heavy traffic [25,26].

**Figure 7:**
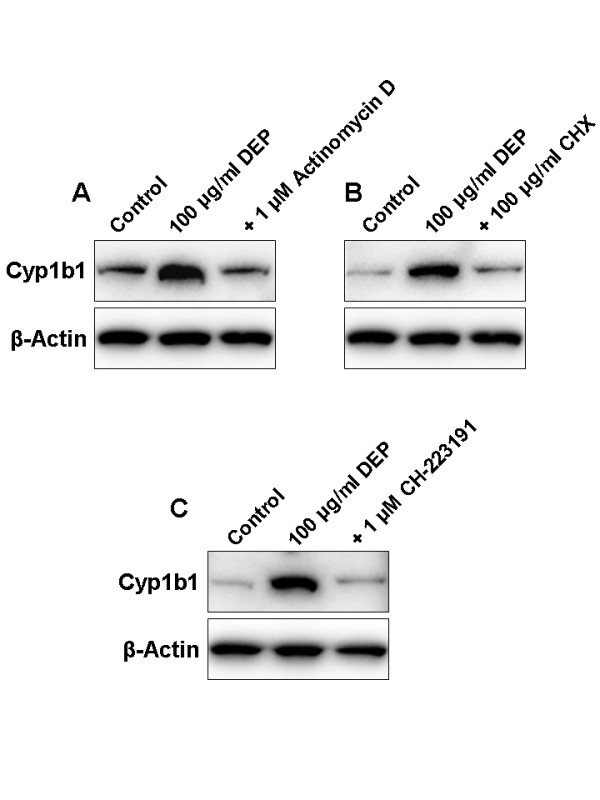
**DEP-mediated Cyp1b1 upregulation involves AhR**. Western blots showing Cyp1b1 protein expression; β-actin was used as loading control. DEP-mediated upregulation of Cyp1b1 protein expression in isolated rat brain microvessels is blocked with (A) actinomycin D, an inhibitor of transcription, (B) cycloheximide (CHX), an inhibitor of protein synthesis, and (C) an AhR antagonist (CH-223191).

We also addressed the mechanism of DEP-mediated Cyp1b1 induction in rat brain microvessels. Inhibiting transcription with actinomycin D or inhibiting protein synthesis with cycloheximide (CHX) abolished DEP-mediated upregulation of Cyp1b1 protein expression in isolated rat brain microvessels (Figures 7A and 7B). Importantly, the use of the AhR antagonist CH-223191 blocked this DEP-mediated Cyp1b1 induction in brain microvessels (Figure 7C), indicating that this effect is AhR-dependent. Thus, these data demonstrate that DEP induces Cyp1b1 in brain microvessels *ex vivo *and *in vivo *and that this effect is likely to be AhR-dependent.

## Discussion

In peripheral tissues such as liver, CYP enzymes are regulated by the same transcription factors including PXR, CAR and AhR [2]. However, few studies exist on the regulatory pathways of these enzymes at the BBB. Recently, we demonstrated that AhR transcripts are abundant in human brain microvessels [1] and that TCDD, the highly potent AhR ligand, strongly upregulated *CYP1A1 *and *CYP1B1 *in hMEC/D3 cells, a promising *in vitro *model of the human BBB [11]. Based on these findings, we tested if AhR pathway is involved *in vivo *in the regulation of Cyp1b1 in rat brain microvessels. The present results show for the first time, AhR expression and immunostaining in rat brain microvessels in addition to Cyp1b1 upregulation after *in vivo *and *ex vivo *exposure to well-known AhR ligands: TCDD and heavy hydrocarbons-containing DEP. In this way, rat brain microvessels are a critical target of AhR environmental pollutant ligands.

We first focused on AhR expression and localisation in isolated rat brain microvessels. AhR was highly expressed at both mRNA and protein level in rat brain microvessels. These findings are in accordance with the study of Filbrandt *et al*. showing that isolated murine cerebral vascular endothelial cells express abundant level of AhR protein [14]. By immunostaining, we localised AhR protein within the cytoplasm of rat brain microvessel endothelial cells corresponding to the predominant distribution of inactive (not ligand-bound) AhR [9]. Regarding Cyp1b1, expression of this enzyme at the BBB is controversial. Cyp1b1 was not detected at the mouse BBB but was immunolocalised in other BBB interfaces like the leptomeninges [12]. Furthermore, basal protein expression was undetected in primary murine vascular endothelial cells [14] whereas CYP1B1 has been localised in human brain capillaries by immunohistochemistry [32]. Here we found that Cyp1b1 is constitutively expressed in different extrahepatic rat tissues including brain and especially in brain microvessels. Our western blot analysis of Cyp1b1 expression showed that the apparent molecular weight slightly differed depending on tissue samples suggesting that transcriptional and/or post-transcriptional/translational events might affect Cyp1b1 molecular weight. To our knowledge, alternatively Cyp1b1 spliced variants have not been described in the literature in contrast to Cyp1a1 ones reported in human brain [33].

Thus, like in human BBB, rat brain microvessels express both Cyp1b1 and AhR, being therefore a suitable model to assess AhR regulation pathway *in vivo *at this interface.

We then studied TCDD effect on AhR-target gene *Cyp1b1 *expression in rat brain microvessels. Here, we report that Cyp1b1 was strongly induced by TCDD as early as 3 h after exposing *ex vivo *rat brain microvessels to 25 nM TCDD. In *in vivo *experiments, TCDD induced an early and sustained upregulation of Cyp1b1 protein expression in rat brain microvessels. In fact Cyp1b1 protein expression induction occurred at 12 h post-injection and was maintained throughout 72 h. Our results are consistent with a very recent study showing that acute TCDD treatment upregulates Cyp1b1 at the rat BBB 48 hours post-injection [34]. In our study, *in vivo *Cyp1b1 induction happened earlier (12 h after TCDD single dose) allowing less time for potential indirect mechanisms to occur.

TCDD also induced Cyp1a1 expression at the mRNA level *ex vivo *and *in vivo*. Despite high induction, we could not detect Cyp1a1 protein in isolated brain microvessels from control or TCDD-treated rats. By qPCR we found in brain microvessels from naïve rats substantially lower *Cyp1a1 *mRNA levels compared to *Cyp1b1 *mRNA levels (60-fold difference). Thus, *Cyp1a1 *mRNA is barely detectable, which is consistent with findings in human brain microvessels [1]. Consequently, Cyp1a1 protein was also undetectable in rat brain microvessels.

However, Wang et al. recently detected Cyp1a1 protein expression in isolated rat brain microvessels which was increased by TCDD [34]. This suggests that discrepancies between studies may come from different technical approaches. However, using ultracentrifugation Wang et al. isolated brain microvessel crude membranes that include those of the endoplasmic reticulum where Cyp1a1 is localised, thus allowing Cyp1a1 enrichment and subsequent detection by Western blotting.

Δ^9^-THC has been reported to increase *Cyp1a1 *mRNA in murine Hepa-1 cells through AhR pathway [17]. In the present study, we did not observe induction in *Cyp1a1 *and *Cyp1b1 *expression in rat brain microvessels after sub-chronic Δ^9^-THC treatment, which is consistent with our previous results showing an absence of *CYP1A1 *and *CYP1B1 *induction after Δ^9^-THC exposure of the human cerebral endothelial cell line hCMEC/D3 [11]. This discrepancy with experiment of Roth *et al*. [17], may be due to inter-species differences or to a difference in the dosing range. Indeed, pharmacokinetic studies showed that after sub-chronic Δ^9^-THC treatment of rats, plasma concentrations of Δ^9^-THC were in the 2-4 ng/mL range [35], which is close to those plasma concentrations observed in humans cannabis abuser [36] and about three orders of magnitude lower than the Δ^9^-THC concentrations needed to induce Cyp1a1 *in vitro *[17]. Through marijuana consumption, users are exposed to psychoactive cannabinoids and also to toxicant-containing tar such as PAHs [37]. These AhR ligands have been reported to occur at higher levels in marijuana than in tobacco-tar [38]. Thus, even if Δ^9^-THC does not activate AhR in rats *in vivo*, PAHs-containing marijuana tar could activate AhR. Given the widespread use of marijuana around the world, further experiments are needed to assess potential AhR activation by marijuana tar at the BBB.

DEP are complex environmental toxicants consisting of a central core of elemental carbon and adsorbed organic compounds such as PAHs and HAHs [18,19]. Here we observed that a chronic inhalation exposure to DEP strongly upregulated Cyp1b1 expression *in vivo *in rat brain microvessels. *Ex vivo*, DEP also induced Cyp1b1 expression in rat brain microvessels. Furthermore, this effect was abolished by blocking transcription or translation or by using an AhR antagonist, strongly suggesting that DEP-mediated Cyp1b1 upregulation is triggered by AhR activation.

AhR involvement in toxicological responses and carcinogenesis has been widely studied in the last decades. However, increasing evidence suggests that AhR is not only a cellular sensor of environmental pollutants but also a key regulator of physiological functions. In fact, AhR is involved in reproduction, immunity, and a wide variety of basic cellular processes including proliferation, migration, adhesion, and differentiation [9,10]. In addition, AhR^-^/^- ^mice display abnormal vascular structures in kidney, liver, and eyes, which suggests that AhR is involved in vascular development [39]. Recently, the highly potent AhR ligand TCDD has been shown to decrease cerebral blood flow and alter brain vessel morphology in developing zebrafish, suggesting that TCDD through AhR activation may alter brain vascular physiology [40]. Regarding the physiological function of the Cyp1b1 enzyme, Cyp1b1^-^/^- ^mice exhibit no apparent abnormalities except in their anterior eye segment similar to what has been reported for patients with primary congenital glaucoma [15]. Interestingly, primary cultured retinal endothelial cells from Cyp1b1^-^/^- ^mice lose their ability to undergo capillary morphogenesis [41], suggesting that Cyp1b1 is involved in angiogenesis. Furthermore, Cyp1b1 metabolises several endogenous compounds like retinol, estrogens, and arachidonic acid [7], which is turned into epoxyeicosatrienoic acid that has vasodilatatory properties [42]. Interestingly, 17-β-estradiol, a substrate of CYP1B1, increased the expression of vasoactive factors in HUVEC [43]. Taken together, these data indicate that both AhR and Cyp1b1 could be key elements in the development and/or maintenance of a functional BBB.

Besides, Cyp1b1 is known to bioactivate procarcinogens such as PAHs into more reactive metabolites so that its induction may alter the BBB [16]. Through activating AhR, PAHs induce CYP1B1 expression and in turn promote their own activation into carcinogens. Therefore, DEP induction of CYP1B1 at the BBB may bioactivate PAHs adsorbed to DEP into genotoxic intermediates [44,45] that could increase levels of potentially harmful chemicals, which may initiate BBB dysfunction and/or brain disease.

## Conclusion

The present study has shown Cyp1b1 upregulation by environmental pollutants TCDD and DEP *in vivo *in rat brain microvessels. Since BBB is likely to be a target of AhR environmental pollutant ligands, further experiments are required to assess the role AhR/Cyp1b1 play at the BBB, especially with regard to their impact in toxicological and/or pathophysiological processes. It will therefore be critical to explore whether the AhR/Cyp1b1 in the brain microvessel endothelium is beneficial or harmful to BBB function.

## Competing interests

This research was supported by UMN CoP start-up funds (to BB).

## Authors' contributions

AJ and AH have carried out all the experiments and written the manuscript. SY and SP have been involved in drafting the manuscript. XC, BB, and JMS have been involved in critically revising the manuscript. XD conceived the design of the study and coordinate drafting the manuscript. All authors have read and approved the final version of this manuscript.

## References

[B1] Dauchy S Dutheil F Weaver RJ Chassoux F Daumas-Duport C Couraud PO Scherrmann JM De Waziers I Decleves X ABC transporters, cytochromes P450 and their main transcription factors: expression at the human blood-brain barrier J Neurochem 2008 107 1518 1528 10.1111/j.1471-4159.2008.05720.x 19094056

[B2] Xu C Li CY Kong AN Induction of phase I, II and III drug metabolism/transport by xenobiotics Arch Pharm Res 2005 28 249 268 10.1007/BF02977789 15832810

[B3] Denison MS Nagy SR Activation of the aryl hydrocarbon receptor by structurally diverse exogenous and endogenous chemicals Annu Rev Pharmacol Toxicol 2003 43 309 334 10.1146/annurev.pharmtox.43.100901.135828 12540743

[B4] Denison MS Pandini A Nagy SR Baldwin EP Bonati L Ligand binding and activation of the Ah receptor Chem Biol Interact 2002 141 3 24 10.1016/S0009-2797(02)00063-7 12213382

[B5] Swanson HI DNA binding and protein interactions of the AHR/ARNT heterodimer that facilitate gene activation Chem Biol Interact 2002 141 63 76 10.1016/S0009-2797(02)00066-2 12213385

[B6] Kawajiri K Fujii-Kuriyama Y Cytochrome P450 gene regulation and physiological functions mediated by the aryl hydrocarbon receptor Arch Biochem Biophys 2007 464 207 212 10.1016/j.abb.2007.03.038 17481570

[B7] Nebert DW Dalton TP The role of cytochrome P450 enzymes in endogenous signalling pathways and environmental carcinogenesis Nat Rev Cancer 2006 6 947 960 10.1038/nrc2015 17128211

[B8] Ebert B Seidel A Lampen A Identification of BCRP as transporter of benzo[a]pyrene conjugates metabolically formed in Caco-2 cells and its induction by Ah-receptor agonists Carcinogenesis 2005 26 1754 1763 10.1093/carcin/bgi139 15917307

[B9] Barouki R Coumoul X Fernandez-Salguero PM The aryl hydrocarbon receptor, more than a xenobiotic-interacting protein FEBS Lett 2007 581 3608 3615 10.1016/j.febslet.2007.03.046 17412325

[B10] Puga A Ma C Marlowe JL The aryl hydrocarbon receptor cross-talks with multiple signal transduction pathways Biochem Pharmacol 2009 77 713 722 10.1016/j.bcp.2008.08.031 18817753 PMC2657192

[B11] Dauchy S Miller F Couraud PO Weaver RJ Weksler B Romero IA Scherrmann JM De Waziers I Decleves X Expression and transcriptional regulation of ABC transporters and cytochromes P450 in hCMEC/D3 human cerebral microvascular endothelial cells Biochem Pharmacol 2009 77 897 909 10.1016/j.bcp.2008.11.001 19041851

[B12] Granberg L Ostergren A Brandt I Brittebo EB CYP1A1 and CYP1B1 in blood-brain interfaces: CYP1A1-dependent bioactivation of 7,12-dimethylbenz(a)anthracene in endothelial cells Drug Metab Dispos 2003 31 259 265 10.1124/dmd.31.3.259 12584151

[B13] Huang P Rannug A Ahlbom E Hakansson H Ceccatelli S Effect of 2,3,7,8-tetrachlorodibenzo-p-dioxin on the expression of cytochrome P450 1A1, the aryl hydrocarbon receptor, and the aryl hydrocarbon receptor nuclear translocator in rat brain and pituitary Toxicol Appl Pharmacol 2000 169 159 167 10.1006/taap.2000.9064 11097868

[B14] Filbrandt CR Wu Z Zlokovic B Opanashuk L Gasiewicz TA Presence and functional activity of the aryl hydrocarbon receptor in isolated murine cerebral vascular endothelial cells and astrocytes Neurotoxicology 2004 25 605 616 10.1016/j.neuro.2003.08.007 15183014

[B15] Vasiliou V Gonzalez FJ Role of CYP1B1 in glaucoma Annu Rev Pharmacol Toxicol 2008 48 333 358 10.1146/annurev.pharmtox.48.061807.154729 17914928

[B16] Shimada T Fujii-Kuriyama Y Metabolic activation of polycyclic aromatic hydrocarbons to carcinogens by cytochromes P450 1A1 and 1B1 Cancer Sci 2004 95 1 6 10.1111/j.1349-7006.2004.tb03162.x 14720319 PMC11158916

[B17] Roth MD Marques-Magallanes JA Yuan M Sun W Tashkin DP Hankinson O Induction and regulation of the carcinogen-metabolizing enzyme CYP1A1 by marijuana smoke and delta (9)-tetrahydrocannabinol Am J Respir Cell Mol Biol 2001 24 339 344 11245634 10.1165/ajrcmb.24.3.4252

[B18] Wichmann HE Diesel exhaust particles Inhal Toxicol 2007 19 Suppl 1 241 244 17886072 10.1080/08958370701498075

[B19] Miyabara Y Hashimoto S Sagai M Morita M PCDDs and PCDFs in vehicle exhaust particles in Japan Chemosphere 1999 39 143 150 10.1016/S0045-6535(98)00595-5 10377969

[B20] Certificate of analysis for standard reference material 2975, diesel particulate matter https://www-s.nist.gov/srmors

[B21] Chopra M Dharmarajan AM Meiss G Schrenk D Inhibition of UV-C light-induced apoptosis in liver cells by 2,3,7,8-tetrachlorodibenzo-p-dioxin Toxicol Sci 2009 111 49 63 10.1093/toxsci/kfp128 19520675

[B22] Brauze D Widerak M Cwykiel J Szyfter K Baer-Dubowska W The effect of aryl hydrocarbon receptor ligands on the expression of AhR, AhRR, ARNT, Hif1alpha, CYP1A1 and NQO1 genes in rat liver Toxicol Lett 2006 167 212 220 10.1016/j.toxlet.2006.09.010 17069994

[B23] Deb S Bandiera SM Characterization of a new cytochrome P450 enzyme, CYP2S1, in rats: its regulation by aryl hydrocarbon receptor agonists Toxicology 2010 267 91 98 10.1016/j.tox.2009.10.025 19883719

[B24] Saxena RK Gilmour MI Schladweiler MC McClure M Hays M Kodavanti UP Differential pulmonary retention of diesel exhaust particles in Wistar Kyoto and spontaneously hypertensive rats Toxicol Sci 2009 111 392 401 10.1093/toxsci/kfp164 19635756

[B25] Kodavanti UP Thomas R Ledbetter AD Schladweiler MC Shannahan JH Wallenborn JG Lund AK Campen MJ Butler EO Gottipolu RR Nyska A Richards JE Andrews D Jaskot RH McKee J Kotha SR Patel RB Parinandi NL Vascular and Cardiac Impairments in Rats Inhaling Ozone and Diesel Exhaust Particles Environ Health Perspect10.1289/ehp.1002386 PMC3059992 20980218

[B26] Risom L Dybdahl M Moller P Wallin H Haug T Vogel U Klungland A Loft S Repeated inhalations of diesel exhaust particles and oxidatively damaged DNA in young oxoguanine DNA glycosylase (OGG1) deficient mice Free Radic Res 2007 41 172 181 10.1080/10715760601024122 17364943

[B27] Hartz AM Bauer B Block ML Hong JS Miller DS Diesel exhaust particles induce oxidative stress, proinflammatory signaling, and P-glycoprotein up-regulation at the blood-brain barrier Faseb J 2008 22 2723 2733 10.1096/fj.08-106997 18474546 PMC2493447

[B28] Yousif S Marie-Claire C Roux F Scherrmann JM Decleves X Expression of drug transporters at the blood-brain barrier using an optimized isolated rat brain microvessel strategy Brain Res 2007 1134 1 11 17196184 10.1016/j.brainres.2006.11.089

[B29] Calderon-Garciduenas L Franco-Lira M Torres-Jardon R Henriquez-Roldan C Barragan-Mejia G Valencia-Salazar G Gonzalez-Maciel A Reynoso-Robles R Villarreal-Calderon R Reed W Pediatric respiratory and systemic effects of chronic air pollution exposure: nose, lung, heart, and brain pathology Toxicol Pathol 2007 35 154 162 10.1080/01926230601059985 17325984

[B30] Calderon-Garciduenas L Maronpot RR Torres-Jardon R Henriquez-Roldan C Schoonhoven R Acuna-Ayala H Villarreal-Calderon A Nakamura J Fernando R Reed W Azzarelli B Swenberg JA DNA damage in nasal and brain tissues of canines exposed to air pollutants is associated with evidence of chronic brain inflammation and neurodegeneration Toxicol Pathol 2003 31 524 538 14692621 10.1080/01926230390226645

[B31] Peters A Veronesi B Calderon-Garciduenas L Gehr P Chen LC Geiser M Reed W Rothen-Rutishauser B Schurch S Schulz H Translocation and potential neurological effects of fine and ultrafine particles a critical update Part Fibre Toxicol 2006 3 13 10.1186/1743-8977-3-13 16961926 PMC1570474

[B32] Rieder CR Parsons RB Fitch NJ Williams AC Ramsden DB Human brain cytochrome P450 1B1: immunohistochemical localization in human temporal lobe and induction by dimethylbenz(a)anthracene in astrocytoma cell line (MOG-G-CCM) Neurosci Lett 2000 278 177 180 10.1016/S0304-3940(99)00932-5 10653022

[B33] Kommaddi RP Turman CM Moorthy B Wang L Strobel HW Ravindranath V An alternatively spliced cytochrome P4501A1 in human brain fails to bioactivate polycyclic aromatic hydrocarbons to DNA-reactive metabolites J Neurochem 2007 102 867 877 10.1111/j.1471-4159.2007.04599.x 17630984

[B34] Wang X Hawkins BT Miller DS Aryl hydrocarbon receptor-mediated up-regulation of ATP-driven xenobiotic efflux transporters at the blood-brain barrier Faseb J 2011 25 644 652 10.1096/fj.10-169227 21048045 PMC3023393

[B35] Nahas GG Frick HC Lattimer JK Latour C Harvey D Pharmacokinetics of THC in brain and testis, male gametotoxicity and premature apoptosis of spermatozoa Hum Psychopharmacol 2002 17 103 113 10.1002/hup.369 12404700

[B36] Grotenhermen F Pharmacokinetics and pharmacodynamics of cannabinoids Clin Pharmacokinet 2003 42 327 360 10.2165/00003088-200342040-00003 12648025

[B37] Moir D Rickert WS Levasseur G Larose Y Maertens R White P Desjardins S A comparison of mainstream and sidestream marijuana and tobacco cigarette smoke produced under two machine smoking conditions Chem Res Toxicol 2008 21 494 502 10.1021/tx700275p 18062674

[B38] Lee ML Novotny M Bartle KD Gas chromatography/mass spectrometric and nuclear magnetic resonance spectrometric studies of carcinogenic polynuclear aromatic hydrocarbons in tobacco and marijuana smoke condensates Anal Chem 1976 48 405 416 10.1021/ac60366a048 1247170

[B39] Lahvis GP Lindell SL Thomas RS McCuskey RS Murphy C Glover E Bentz M Southard J Bradfield CA Portosystemic shunting and persistent fetal vascular structures in aryl hydrocarbon receptor-deficient mice Proc Natl Acad Sci USA 2000 97 10442 10447 10973493 10.1073/pnas.190256997 PMC27043

[B40] Teraoka H Ogawa A Kubota A Stegeman JJ Peterson RE Hiraga T Malformation of certain brain blood vessels caused by TCDD activation of Ahr2/Arnt1 signaling in developing zebrafish Aquat Toxicol 2010 99 241 247 10.1016/j.aquatox.2010.05.003 20554057 PMC3040289

[B41] Tang Y Scheef EA Wang S Sorenson CM Marcus CB Jefcoate CR Sheibani N CYP1B1 expression promotes the proangiogenic phenotype of endothelium through decreased intracellular oxidative stress and thrombospondin-2 expression Blood 2009 113 744 754 10.1182/blood-2008-03-145219 19005183 PMC2628380

[B42] Sudhahar V Shaw S Imig JD Epoxyeicosatrienoic acid analogs and vascular function Curr Med Chem 2010 17 1181 1190 10.2174/092986710790827843 20158473 PMC2855336

[B43] Andersson H Garscha U Brittebo E Effects of PCB126 and 17beta-oestradiol on endothelium-derived vasoactive factors in human endothelial cells Toxicology 2011 285 46 56 10.1016/j.tox.2011.04.003 21513769

[B44] Kumagai Y Arimoto T Shinyashiki M Shimojo N Nakai Y Yoshikawa T Sagai M Generation of reactive oxygen species during interaction of diesel exhaust particle components with NADPH-cytochrome P450 reductase and involvement of the bioactivation in the DNA damage Free Radic Biol Med 1997 22 479 487 10.1016/S0891-5849(96)00341-3 8981040

[B45] Yamazaki H Hatanaka N Kizu R Hayakawa K Shimada N Guengerich FP Nakajima M Yokoi T Bioactivation of diesel exhaust particle extracts and their major nitrated polycyclic aromatic hydrocarbon components, 1-nitropyrene and dinitropyrenes, by human cytochromes P450 1A1, 1A2, and 1B1 Mutat Res 2000 472 129 138 11113705 10.1016/s1383-5718(00)00138-8

